# Alterations in the pH of pancreatic juice are associated with chymotrypsin C inactivation and lithostathine precipitation in chronic pancreatitis patients: a proteomic approach

**DOI:** 10.1186/s12014-022-09384-8

**Published:** 2022-12-26

**Authors:** Renuka Goudshelwar, Bala Manikanta Adimoolam, Sundeep Lakhtakia, Jagadeshwar Reddy Thota, Prabhakar Sripadi, Karuna Rupula, D Nageshwar Reddy, Mitnala Sasikala

**Affiliations:** 1 Biochemistry Labs, Translational Research Centre, Asian Healthcare Foundation, AIG Hospitals, Gachibowli, Hyderabad, 500032 Telangana India; 2 Department of Medical Gastroenterology, AIG Hospitals, Gachibowli, Hyderabad, 500032 Telangana India; 3 Center for Mass Spectrometry, CSIR–Indian Institute Of Chemical Technology, Uppal Rd, IICT Colony, Tarnaka, Hyderabad, 500007 Telangana India; 4 Department of Biochemistry, University College of Science, Osmania University, Osmania University Main Rd, Hyderabad, 500007 Telangana India

**Keywords:** ERCP, Lithostathine, MALDI-TOF, Pancreatic juice, Protein plugs

## Abstract

**Background:**

The progression of chronic pancreatitis (CP), an inflammatory disease of the pancreas, causes pancreatic stones to form within the pancreatic ductal lumen/parenchyma, which occurs via protein plug formation. Pain is the most common symptom that necessitates clinical attention, and pain relief is the therapeutic goal for these patients. Endoscopic therapy and surgery are complimentary forms of therapy for pain relief. This study was envisaged to clarify the mechanism by which protein plug/soft stones form in pancreatic ducts prior to undergoing calcification.

**Methods:**

Protein plugs were obtained from twenty CP patients undergoing therapeutic ERCP for stone removal. Pancreatic juice was obtained from five CP patients without stones. Proteins were isolated by TCA/acetone precipitation, SDS PAGE and 2-D gel electrophoresis to determine the protein profile. Protein spots from the 2-D gel were excised and subjected to matrix-assisted laser desorption/ionization-time of flight (MALDI-TOF) for identification. The effect of altered pH and elevated concentrations of trypsin on pancreatic juice protein was assessed by SDS‒PAGE to determine the protein profile. Differentially expressed protein bands were excised and subjected to MALDI-TOF. In silico analysis was performed by docking lithostathine with the calcite molecule using AutoDock Vina and PyMOL to clarify their interaction during stone formation.

**Results:**

Twenty-three and twenty-nine spots from 2D gels of protein plugs and pancreatic juice, respectively, revealed that lithostathine (Reg1A) was the only protein in the protein plugs, whereas digestive enzymes and lithostathine were identified in pancreatic juice. Altered pH levels and increased trypsin concentrations in the pancreatic juice caused a protein to degrade via an unknown mechanism, and this protein was identified as chymotrypsin C (CTRC) by MALDI-TOF. Docking studies showed that the binding affinity of calcite was higher with the cleaved lithostathine, explaining the deposition of calcium that was observed around the protein plugs after calcified stones were formed through precipitation.

**Conclusion:**

Our results suggest that chymotrypsin C (CTRC) is degraded in an acidic environment, leading to the precipitation of lithostathine in the ductal lumen.

**Graphical Abstract:**

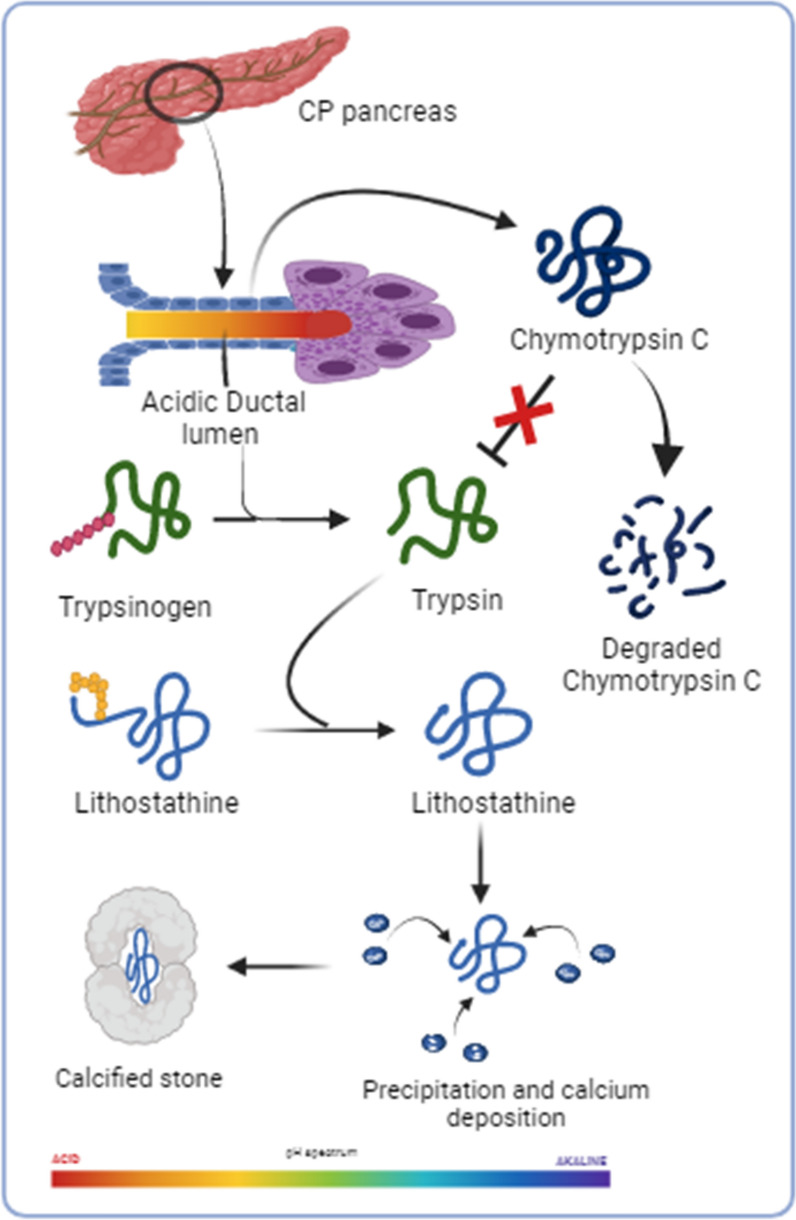

**Supplementary Information:**

The online version contains supplementary material available at 10.1186/s12014-022-09384-8.

## Introduction

Chronic pancreatitis (CP) is a progressive fibroinflammatory disease [1] caused by various aetiological factors, such as alcohol, hereditary, idiopathic, obstruction and autoimmunity, and eventually leads to exocrine and endocrine deficiencies [2]. As the disease progresses from an acute stage to chronicity, insoluble proteins aggregate within the lumen of the pancreatic duct [3]. Calcification of these proteins within the main pancreatic duct leads to calcific chronic pancreatitis [4]. When these calcifications or stones form within the main pancreatic duct, intraductal pressure becomes elevated because the flow of pancreatic juice is blocked, causing these patients intense and unbearable pain [5]. It is known that in Southeast Asian countries, pancreatic ductal stones are large in patients with chronic pancreatitis. The incidence of diabetes associated with exocrine pancreatic disease was reported to be higher when calcifications are present [6].

Several studies have demonstrated that ductal cell dysfunction during inflammation in CP results in low bicarbonate secretion [7]. Bicarbonate plays a crucial role in neutralizing the acidic contents secreted by acinar cells and maintaining the alkaline pH of the ductal lumen. Lowered bicarbonate secretion leads to an imbalance in the regulation of pH within the ductal lumen. It is hypothesized that a lower pH leads to protein precipitation (protein plug formation) [8]. Earlier studies demonstrated that the major component of the proteinaceous core of soft stone/protein plugs is composed of a protein, which was initially termed pancreatic stone protein (PSP) and later renamed lithostathine S (soluble, secretory form) and lithostathine H (insoluble form) [9]. Premature activation of trypsinogen to the activated form causes the N-terminal 11 amino acid sequence to cleave the soluble form of lithostathine, converting it into an insoluble form; the protein precipitates and aggregates as a result, then eventually forms protein plugs in the pancreas [10]. The mechanism that leads to protein plug/stone formation has not yet been investigated. Therefore, the sequence of events from inflammation to protein aggregation in the pancreatic ducts of CP patients urgently needs to be clarified. Hence, this study was performed to clarify the protein plug composition by performing proteomic profiling of protein plugs/soft stones and to study the mechanism involved in protein plug/soft stone formation.

## Study design

This is a prospective study, and the protein plug/soft stone was collected from January 2021 to March 2021. Patients with idiopathic chronic pancreatitis (CP) that were identified to have calcified stones, protein plugs or soft stones on EUS (endoscopic ultrasound) were recruited. Protein plugs were collected during endoscopic retrograde cholangiopancreatography (ERCP) and subjected to protein profiling employing MALDI-TOF (matrix-assisted laser desorption ionization time-of-flight) mass spectrometry to identify proteins in the soft stones. Pancreatic juice was collected from CP patients with external pancreatic fistula without protein plugs, and the samples were subjected to alterations in pH to mimic ductal pH changes under elevated activated trypsin concentrations in CP; this was performed to observe protein precipitation and aggregation. In silico analysis was performed to estimate the affinity of the protein towards calcium.

## Materials and methods

### Soft stone/protein plug collection

The soft stones were extracted during the standard ERCP technique using accessories such as Roth net or polyp traps. The stones were retrieved in PBS (phosphate buffered saline) and stored at − 80 °C for protein isolation and analysis.

### Pancreatic juice collection

Pancreatic juice was obtained from CP patients with grossly dilated PD (pancreatic duct), as established from prior imaging. During therapeutic ERCP, pancreatic juice was initially aspirated using a sphincterotome or cannula selectively passed into the PD, followed by definitive endoscopic therapy. The juice was placed into a sterile falcon tube containing a protease inhibitor and stored at − 80 °C for further analysis.

### Isolating the proteins from soft stones

The stones were collected in 5 mL PBS and concentrated to a final volume of 1 mL using a Speed-Vac (ScanVac Coolsafe, Labogene, Scandinavian by design, Lillerod, Denmark). Then, 100 µL of 100% TCA was added to the stones, which were incubated on ice for 30 min. After incubation, 50 mM NaCl and 4 volumes of acetone (100%) were added to the solution and incubated overnight at − 20 °C. The samples were centrifuged at 20,000 ×*g* for 30 min. The supernatant was removed carefully, and the pellet was air dried for 10 min. The pellet was then resuspended in 1 M Tris (pH 7.2) and quantitated using a Nano drop. The remaining protein solution was stored at − 20 °C until further analysis.

### 2D Gel electrophoresis

Protein solution (2 mg protein dissolved in 1 M Tris; pH 7.2) was used to precipitate protein by adding 4 volumes of acetone and incubating the solution at − 20 °C overnight. The samples were centrifuged at 10,000*g* at 4 °C for 30 min. The pellet was washed twice with ice-cold acetone and air dried. Two-dimensional gel electrophoresis was carried out following the protocol described by Sengupta et al*.* [11]. The pellet was dissolved in 320 µL of rehydration buffer and incubated at room temperature overnight to dissolve the protein. After incubation, 0.004% of Bromophenol Blue indicator dye was added to the solution. The Immobiline DryStrip gels strip (7 cm, pH 4–7, linear gradient; GE Healthcare) was placed in a tray that contained the protein for 30 min. IEF was performed at 20 °C using the following parameters [50 V for 3 h, 150 V 1 h, 250 V for 1 Hr, 500 V for 3 h, increase from 500 V to 10,000 V for 3:50 h, 7 h 10,000 V (a total of 70,000 V h^−1^) hold at 500 V]. After IEF, the strips were placed on 12% SDS PAGE gel and sealed with 0.5% agarose containing trace amounts of bromophenol blue. The gel was stained with Coomassie Brilliant Blue R-250 overnight on an orbital shaker and then destained with distilled water. The gel image was documented in a typhoon image scanner [12, 13].

### In-gel digestion

In-gel digestion was performed following the protocol described by Shevchenko et al*.* [14] with a few modifications. Gel pieces, pretreated with DTT and iodoacetamide, were digested with trypsin (20 ng/µL) [Porcine-Mass Spectrometry grade, G- Biosciences] and incubated overnight at 37 °C. The tubes were centrifuged at 12,000 rpm for 15 min at 4 °C. The supernatant was collected in a fresh tube. Sixty microlitres of extraction buffer (0.1% TFA in 50% acetonitrile) was added to the gel pieces and vortexed for 5 min. The supernatant was collected and subjected to speed-vac to lyophilize the proteins. The dried protein pellet was then resuspended in 7–10 µL of the extraction buffer and analysed by MALDI-TOF.

## MALDI-TOF

### Chemicals and reagents

LC − MS CHROMASOLV grade acetonitrile (ACN) trifluoroacetic acid (TFA) and α-cyano-4-hydroxycinnamic acid (HCCA) as a MALDI matrix were procured from Sigma‒Aldrich (Bangalore, India). Deionized water was obtained from a Milli-Qapparatus (Millipore, Bedford, MA, USA).

### Instrumentation

The MALDI-MS experiment was performed using a MALDI-reTOF/TOF UltrafleXtreme (Bruker Daltonics, Bremen, Germany) fitted with pulsed ion extraction (PIE), reflector lens (re), and LIFT devices. For lower Mw samples (below 4000 Da), high resolution was achieved using the time-of-flight mode combined with a reflector mirror (noted as reTOF) in the PIE device, and for higher Mw samples (beyond 4000 Da), the direct time-of-flight mode was applied (as TOF). Desorption/ionization was achieved using a 2 kHz smart beam II laser (Nd:YAG laser at a wavelength of 355 nm with a pulse duration of 3 ns and laser fluence from 100 to 120 μJ/cm^2^). Data acquisition and data processing were performed using flex Control 3.3 and flex Analysis 3.3 software. The instrumental conditions employed to analyse molecular species in positive ion and reflectron mode in the m/z range 600 − 4000 were as follows: an Ion Source 1 of 20.00 kV, Ion Source 2 of 17.8 kV, Lens of 7.50 kV, and pulsed ion extraction of 140 ns. The mass spectrometer was calibrated before analysis using the manufacturer’s calibration solution.

### Matrix and sample preparation for MALDI-TOF analysis

The HCCA solution was prepared in 50/50 (v/v) 0.1% TFA/ACN at 10 mg/mL. In-gel tryptic digested samples were analysed employing MALDI-TOF after desalting by a Zip-tip (Sigma) [14]. A 0.5 µL aliquot of CHCA matrix was mixed with 0.5 µL of peptide solution and spotted on a MALDI plate. The sample/matrix composite was dried in air and left at room temperature for crystallization. The positive-ion MALDI mass spectra recorded in reflectron mode. Peptide mass fingerprinting data was processed by using the Swiss-Prot database & MASCOT software to identify the proteins. The mass spectral data of the peptides were searched against the Swiss-Prot database (Swiss-Prot release: 2015_12, entries: 550,116 sequences, UniProt ID: P05451) using the MASCOT search engine. In the search parameters, taxonomy was set to Homo sapiens, carbamidomethyl was selected as a fixed modification, and oxidation was selected as a variable modification and peptide mass tolerance were maintained at ± 0.2 Da with 1 maximum number of cleavages.

### Trypsin digestion & pH alteration of pancreatic juice

It has been reported that trypsin is prematurely activated during chronic pancreatitis. Hence, 10 mL of pancreatic juice was collected in a sterile falcon tube through the drain tube of CP patients without calcified/soft stones and was subjected to higher concentrations of trypsin to clarify its effect on the proteins. From these tubes, 1 mL of pancreatic juice was aliquoted into six Eppendorf tubes and incubated with varying concentrations of trypsin [Porcine-Mass Spectrometry grade, G-Biosciences] (2, 4, 6, 8, 10, 12 µg/mL) for 30 min and 1, 2, 3, 4, 5 h at 37 °C in a water bath to determine the concentration of trypsin that causes precipitation under acidic pH conditions.

During the progression of CP, the ductal lumen remains acidic due to insufficient bicarbonate secretion. Therefore, to mimic this condition, the pH of pancreatic juice was modified to an acidic pH that ranged from 4 to 7 (the pH of the pancreatic juice is 8.0) using 1 N HCl, and the changes in protein profiles were assessed. The samples were centrifuged at 12,000 rpm for 10 min at 4 °C. The supernatant was collected in a fresh tube and concentrated using a speed-vac (ScanVac Coolsafe, Labogene, Scandinavian by design, Lillerod, Denmark). The pellet was resuspended in 1 M Tris pH 7.2. The pellet and supernatant samples were analysed on a 10% SDS PAGE gel. The differentially expressed protein band was subjected to MALDI-TOF to identify the protein.

### In silico analysis

The literature suggests that lithostathine (with undecapeptide) is present in the pancreatic juice of healthy individuals, but no calcification is observed in their pancreatic duct [15, 16]. Hence, to clarify the interactions between the lithostathine present in CP (without undecapeptide) and healthy individuals with calcite molecules*,* in silico analysis was performed. The crystal structure of lithostathine (native and truncated) was obtained from the Protein Data Bank (http://www.rscb.org) [17, 18]. The resolution of truncated lithostathine was 1.55 Å (PDB ID: 1LIT), whereas the resolution of native lithostathine was 1QDD (PDB ID: 1.30 Å). The crystal structure of a calcite molecule, which was used as a ligand, was retrieved from the Pubchem database (https://pubchem.ncbi.nlm.nih.gov/), and the Pubchem ID is 516889. Docking of lithostathine with the calcite molecule was carried out to clarify their interactions by using AutoDock Vina and PyMOL provides information in the form of binding energy.

### Statistical analysis

The HbA1c values of CP patients with protein plugs and calcification are expressed as the median and interquartile range (IQR) using box and whisker plots. The clinical parameters across the patients were evaluated by one-way ANOVA and the chi-square test for statistical significance (P < 0.05).

## Results

### Clinical characteristics of patients

**Fig. 1:**
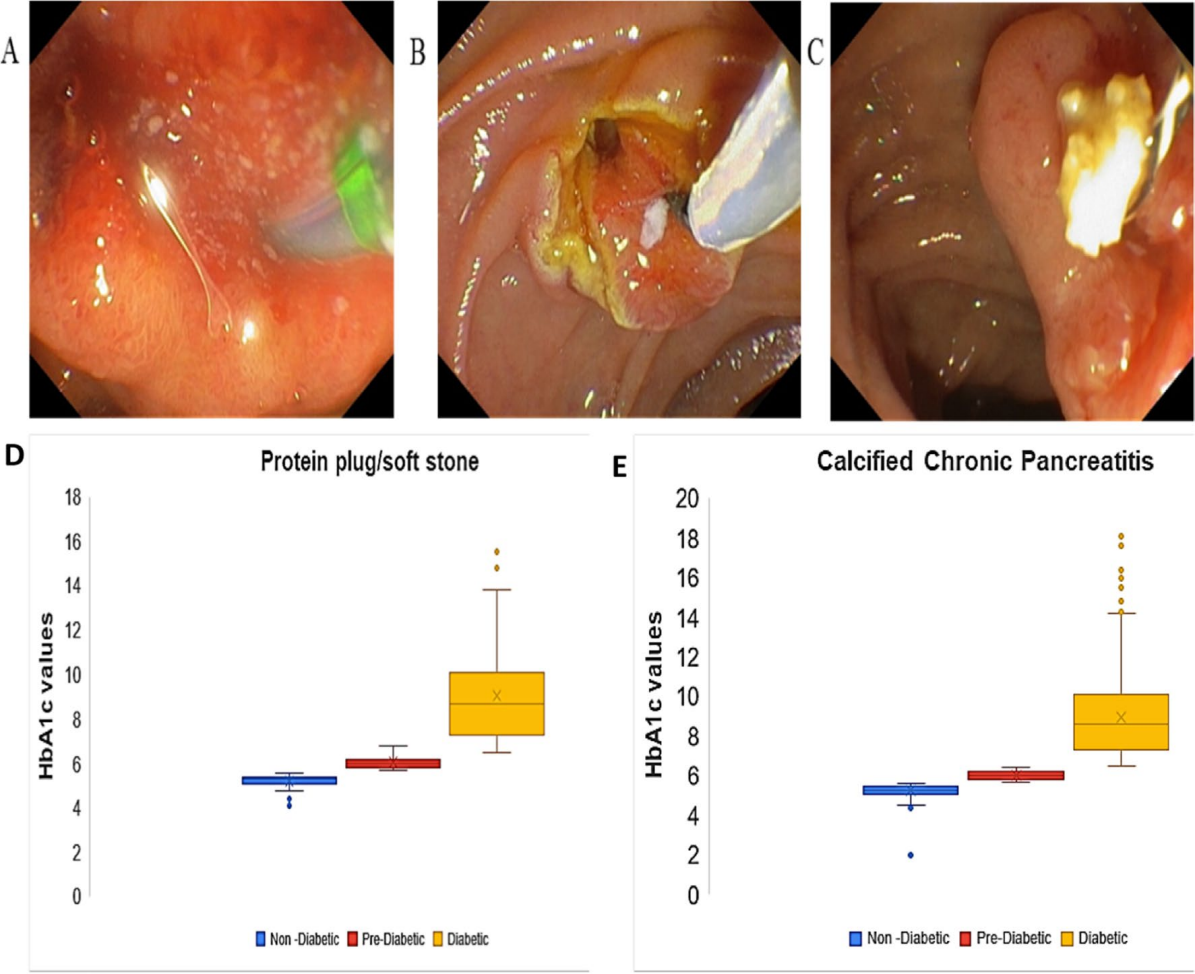
ERCP images: **A** protein plug, **B** radiolucent stone, **C** calcified stone and HbA1c status: **D** box and whisker plot of HbA1c values of CP patients with both soft and calcified stones. (E) Box and whisker plot of HbA1c values of CP patients with calcified stones

All patients diagnosed with CP [n = 2824, age 35.6 ± 13.8 years; male 2024 (72.2%)] were screened for the presence of calcified/soft stones by imaging modalities such as ERCP and endoscopic ultrasonography (Fig. 1 A,B,C). The clinical parameters, along with their significance for CP patients without stones, with protein plugs and calcified chronic pancreatitis are provided in Table 1. A total of 1924 patients (68%) had calcifications/soft stones and 900 (32%) CP patients did not have calcifications/soft stones. Out of 1924, 1678 (59.0%) patients had calcified stones, and 246 patients (41.0%) had both soft stones and calcified stones. The prevalence of soft stones/protein plugs was higher in males (n = 192, 78%; 35.9 ± 13.8 years) compared to females (n = 54, 22%; 27.9 ± 13.5 years): P ≤ 0.0001. Lymphocytes were significantly high in CP patients with protein plugs and calcified stones, while neutrophils were high in CP patients without any stones. HbA1c values above 5.7, indicating a pre-diabetic (PD) status, and above 6.4, indicating a diabetic (D) status, were more common in CP patients in which all stones were calcified (PD: 23%, D: 57%) compared to CP patients with soft and calcified stones (PD: 22%, D: 54%) (Fig. 1D, E).

**Table 1:** Clinical parameters of CP patients without stones, CP patients with protein plugs and patients with calcified chronic pancreatitis

Clinical parameter	CP patients without stones	CP patients with protein plugs	CP patients with calcified stones	P value
Age (years)	36 ± 15 (n = 900)	34 ± 14 (n = 246)	35.7 ± 12.99 (n = 1678)	0.254032571
Gender				
Male	728 (80.89%)	192 (78.05%)	1125 (67%)	** < 0.0001**
Female	172 (19.11%)	54 (21.95%)	553 (33%)	** < 0.0001**
Complete blood picture (CBP)				
Haemoglobin (gm/dL)	12.6 ± 6.5 (n = 460)	12.8 ± 1.9 (n = 177)	12.6 ± 2.05 (n = 734)	0.832539986
Total WBC (cells/mm^3^)	8808.2 ± 4791.4 (n = 460)	8906.8 ± 7655.9 (n = 177)	8105.7 ± 2741.71 (n = 734)	**0.003318517**
Neutrophils (%)	65.7 ± 11.3 (n = 460)	63.5 ± 10.6(n = 177)	62.7 ± 10.14 (n = 734)	**4.68E-09**
Lymphocytes (%)	25.9 ± 10.4 (n = 460)	28.2 ± 9.5 (n = 177)	28.8 ± 9.28 (n = 734)	**2.37227E-10**
Eosinophils (%)	3.1 ± 2.8 (n = 460)	3.3 ± 2.8 (n = 177)	3.2 ± 2.53 (n = 734)	0.469180491
Monocytes (%)	5.3 ± 2.9 (n = 460)	5.0 ± 1.8 (n = 177)	5.3 ± 3.1 (n = 734)	0.160235454
Platelet count (lakhs/mm^3^)	2.6 ± 1.5 (n = 460)	2.5 ± 0.9 (n = 177)	2.4 ± 0.93 (n = 734)	**0.00644478**
Renal function test				
Blood urea (mg/dL)	28.2 ± 18.9 (n = 234)	26.5 ± 23.4 (n = 115)	22.9 ± 11.24 (n = 502)	**1.21441E-06**
Creatinine (mg/dL)	1 ± 0.6 (n = 234)	0.99 ± 0.71 (n = 115)	0.95 ± 1.04 (n = 501)	0.1155404
Liver function test				
Total Bilirubin (mg/dL)	1.2 ± 1.8 (n = 470)	1.01 ± 1.01 (n = 179)	1.2 ± 3.73 (n = 742)	0.291647286
SGPT (ALT) (U/L)	43 ± 63.2 (n = 470)	45.82 ± 66.60(n = 179)	35.4 ± 39.88 (n = 742)	**0.003183362**
SGOT (AST) (U/L)	38.2 ± 50.9 (n = 470)	40.51 ± 62.7 (n = 179)	32.8 ± 28.62 (n = 742)	**0.011630782**
ALP (U/L)	151.5 ± 182.3 (n = 470)	148.7 ± 152.6 (n = 179)	126.9 ± 117.52(n = 742)	**0.002823298**
Total proteins (gm/dL)	7.8 ± 5.2 (n = 470)	7.8 ± 4.4 (n = 179)	7.5 ± 0.74 (n = 742)	0.26545353
Albumin (gm/dL)	4.3 ± 1.3 (n = 470)	4.3 ± 0.6 (n = 179)	4.2 ± 0.58 (n = 742)	0.355579422
Globulin (gm/dL)	3.2 ± 1.4 (n = 470)	3.2 ± 0.5 (n = 179)	3.3 ± 0.5 (n = 742)	0.449561731
Thyroid profile				
T3 (ng/mL)	1.2 ± 1.5 (n = 44)	1.0 ± 0.2 (n = 18)	1.0 ± 0.2 (n = 205)	0.061662891
T4 (µg/dL)	10.3 ± 13.8 (n = 44)	8.1 ± 1.8 (n = 19)	7.8 ± 1.58 (n = 205)	**0.031168078**
TSH (mIU/mL)	3.5 ± 7.4 (n = 98)	2.6 ± 1.5 (n = 32)	2.6 ± 2.87 (n = 254)	**0.02533354**
Lipid profile				
Total Cholesterol (mg/dL)	180.3 ± 48.3 (n = 138)	179.3 ± 41.8 (n = 38)	182.9 ± 44.89 (n = 143)	0.84439935
HDL Cholesterol (mg/dL)	37.3 ± 8.9 (n = 138)	39.8 ± 9.1 (n = 38)	41.7 ± 13.22 (n = 143)	**0.002427905**
VLDL Cholesterol (mg/dL)	32.4 ± 16.3 (n = 138)	29.2 ± 15.0 (n = 38)	31.1 ± 17.79 (n = 143)	0.487241088
Direct LDL Cholesterol (mg/dl)	109 ± 42 (n = 138)	111.1 ± 32.9 (n = 38)	112.7 ± 41.91 (n = 143)	0.734537519
Triglycerides (mg/dL)	166.7 ± 95.9 (n = 138)	146.4 ± 74.3 (n = 38)	153.2 ± 79.85 (n = 155)	0.19558935
Serum Amylase (U/L)	229.4 ± 383.5 (n = 179)	361.1 ± 2027.9 (n = 81)	112.8 ± 232.93 (n = 273)	**0.027901594**
Lipase (U/L)	237 ± 666 (n = 322)	246.8 ± 907.6 (n = 126)	89.1 ± 252.06 (n = 398)	**0.000378482**
Fasting blood glucose (mg/dL)	125.8 ± 60.1 (n = 334)	135.4 ± 94.5 (n = 133)	137.7 ± 71.1 (n = 476)	**0.011276096**
Random blood glucose (mg/dL)	161.9 ± 97.7 (n = 207)	156.1 ± 109.2 (n = 88)	154.9 ± 89.54 (n = 444)	0.566125234
C-peptide (ng/mL)	2.6 ± 2.2 (n = 67)	2.5 ± 2.3 (n = 43)	2.1 ± 2.14 (n = 125)	0.173942407
Erythrocyte sedimentation rate (ESR) (mm/Hr)	30.7 ± 72.7 (n = 255)	24.7 ± 22.1 (n = 99)	27.9 ± 23.35 (n = 474)	0.128745055
Parathyroid hormone (PTH) (pg/mL)	54.9 ± 34.2 (n = 73)	52.7 ± 24.1 (n = 29)	55.4 ± 49.35 (n = 90)	0.940386898
HBA1C %	7.3 ± 2.6 (n = 365)	7.5 ± 2.4 (n = 138)	7.5 ± 2.32 (n = 573)	0.486795981
Vit D (ng/mL)	22.4 ± 21.2 (n = 184)	18.8 ± 10.1 (n = 82)	19.5 ± 12.35 (n = 143)	0.039242099
Vit B12 (pg/mL)	515.7 ± 436 (n = 195)	479.6 ± 432.7(n = 85)	546.6 ± 433.57 (n = 242)	0.365561035
Serum creatinine (mg/dL)	1.01 ± 0.6 (n = 167)	0.9 ± 0.3 (n = 68)	0.96 ± 0.51 (n = 138)	0.214194904
Calcium (mg/dL)	9.5 ± 1.1 (n = 116)	9.5 ± 0.9 (n = 32)	9.7 ± 0.77 (n = 102)	0.25693458
RANSOD (U/mL)	233.3 ± 78.1 (n = 17)	977.7 ± 2386.5 (n = 10)	498.4 ± 1399.1(n = 29)	0.438912
RANSEL (U/L)	8706.3 ± 1517.9 (n = 23)	8792 ± 3688 (n = 11)	8130.6 ± 2128.0 (n = 29)	0.585967
Insulin fasting (MIU/L)	11.3 ± 16.1 (n = 15)	7.29 ± 6.55 (n = 9)	13 ± 34.8 (n = 35)	0.862915
Elastase (Stool) (µg/g)	51.8 ± 13.5 (n = 18)	59.5 ± 43.3 (n = 11)	45.2 ± 34.5 (n = 41)	0.400307

Statistically significant parameters across the disease is given in bold

### Gel electrophoresis analysis of isolated proteins by PAGE and 2-dimensional (2D)

**Fig. 2:**
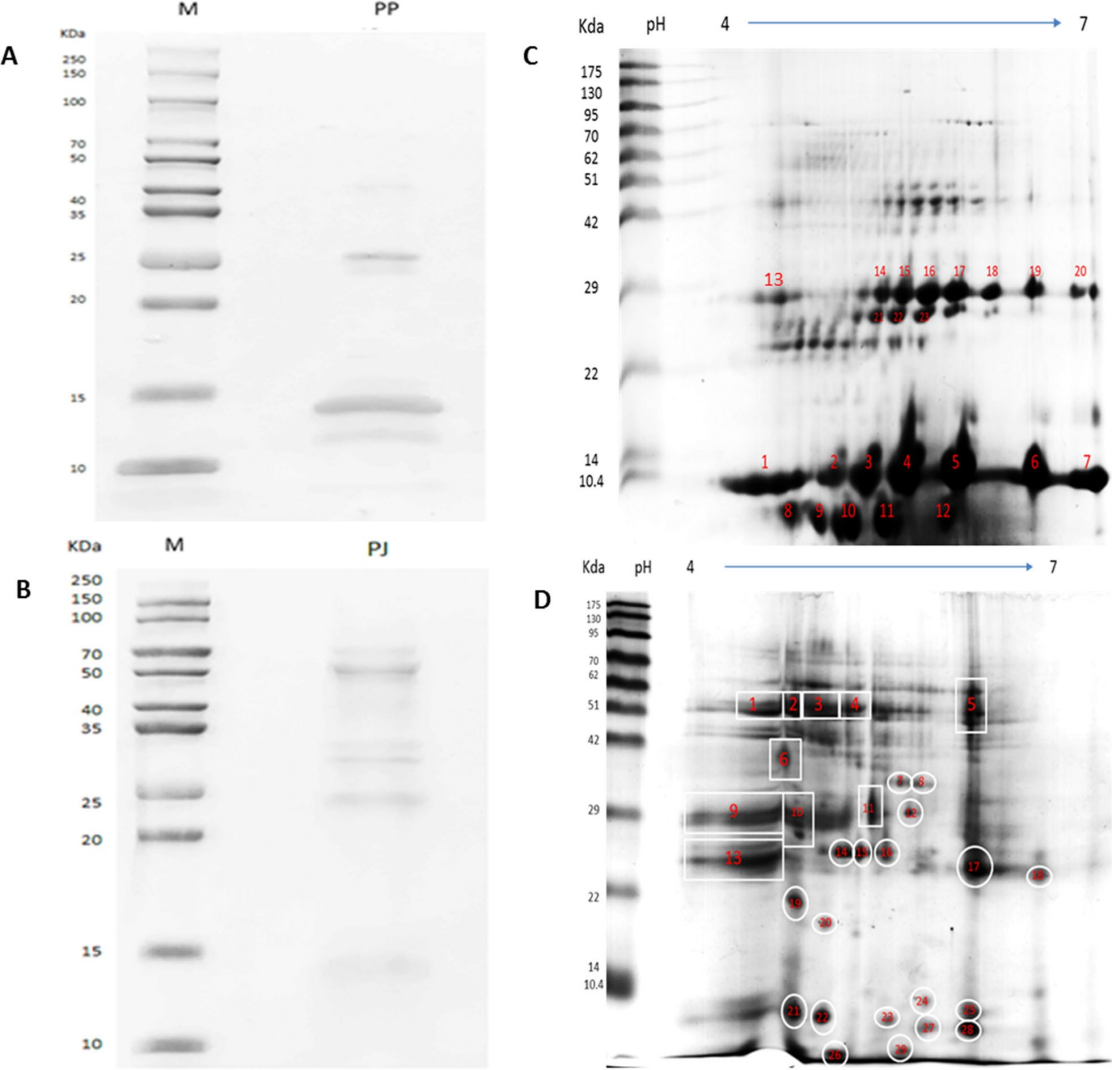
Protein profile: **A** protein plugs and **B** pancreatic juice on a 10% SDS PAGE gel; M is the protein marker. 2-Dimensional gel image of **C** soft stone/protein plugs and **D** pancreatic juice

Pooled analysis of proteins isolated from protein plugs/soft stones showed 2 prominent bands at 14 and 26 kDa regions (Fig. 2A). These samples were then used for 2-dimensional electrophoresis to obtain a better resolution for the protein mixture. The 2D gel (Fig. 2C) showed seven prominent and highly intense spots with similar molecular weights (10.4 kDa) at different PIs ranging from 4 to 7. Five prominent and intense spots were seen at < 10 kDa with PIs ranging between 4.5 and 5.5. Eight tiny but intense spots were seen at 29 kDa with PI ranging between 4 and 7. Three 3 spots were observed at 27 kDa with PI ranging between 5.5 and 6.5. A total of 23 spots with high intensities were chosen for in-gel digestion and identification by MALDI-TOF. Low-intensity spots were excluded because the quantity was not sufficient for MALDI-TOF.

### MALDI-TOF of protein plugs

Mass spectra of peptides obtained after trypsin digestion of twenty-three gel pieces were used to generate the protein mass fingerprint (PMF). The sequences of 14 out of 23 spots of protein plugs matched with Regenerating family member 1 alpha (Reg1A) (60.8%), which is also known as lithostathine. The sequences of all the lithostathine spots had an initial starting position from the 34th amino acid, indicating that the protein cleaved during the process of precipitation and plug formation. Tryptic digestion during in-gel digestion yielded 6 to 11 peptide fragments from the respective 2D gel spots with molecular weights of < 10, 10.4, 27 and 29 kDa and at PIs ranging between 4.17 and 6.97. The Mascot^®^ scores of the identified proteins were between 100 and 170. The sequences of the protein fragments with score greater than 100 is presented in Table 2.

**Table 2:** Peptide mass fingerprint (PMF)

Spot nos	Molecular weight (KDa)	pI (Unit)	Fragments generated	Score	Start–End	Peptide
1	10	4.17	7	125	34–44 34–54 45–54 90–106 90–107 110–122 111–122	ISCPEGTNAYR.S ISCPEGTNAYRSYCYYFNEDR.E SYCYYFNEDR.E ESGTDDFNVWIGLHDPK.K ESGTDDFNVWIGLHDPKK.N RWHWSSGSLVSYK.S WHWSSGSLVSYK.S
4	10	5.34	9	126	34–44 45–54 90–106 90–107 107–109 110–122 111–122 123–148 151–163	ISCPEGTNAYR.S SYCYYFNEDR.E ESGTDDFNVWIGLHDPK.K ESGTDDFNVWIGLHDPKK.N KNR.R RWHWSSGSLVSYK.S WHWSSGSLVSYK.S SWGIGAPSSVNPGYCVSLTSSTGFQK.W DVPCEDKFSFVCK.F
5	10	5.75	10	128	34–44 34–54 45–54 90–106 90–107 107–109 110–122 111–122 123–148 151–163	ISCPEGTNAYR.S ISCPEGTNAYRSYCYYFNEDR.E SYCYYFNEDR.E ESGTDDFNVWIGLHDPK.K ESGTDDFNVWIGLHDPKK.N KNR.R RWHWSSGSLVSYK.S WHWSSGSLVSYK.S SWGIGAPSSVNPGYCVSLTSSTGFQK.W DVPCEDKFSFVCK.F
6	10	6.44	11	163	34–44 34–54 45–54 90–106 90–107 107–109 110–122 111–122 123–148 151–163 158–163	ISCPEGTNAYR.S ISCPEGTNAYRSYCYYFNEDR.E SYCYYFNEDR.E ESGTDDFNVWIGLHDPK.K ESGTDDFNVWIGLHDPKK.N KNR.R RWHWSSGSLVSYK.S WHWSSGSLVSYK.S SWGIGAPSSVNPGYCVSLTSSTGFQK.W DVPCEDKFSFVCK.F FSFVCK.F
7	10	6.97	10	143	34–44 34–54 45–54 90–106 90–107 107–109 110–122 111–122 123–148 151–163	ISCPEGTNAYR.S ISCPEGTNAYRSYCYYFNEDR.E SYCYYFNEDR.E ESGTDDFNVWIGLHDPK.K ESGTDDFNVWIGLHDPKK.N KNR.R RWHWSSGSLVSYK.S WHWSSGSLVSYK.S SWGIGAPSSVNPGYCVSLTSSTGFQK.W DVPCEDKFSFVCK.F
11	< 10	5.15	6	100	34–44 45–54 90–106 90–107 110–122 111–122	ISCPEGTNAYR.S SYCYYFNEDR.E ESGTDDFNVWIGLHDPK.K ESGTDDFNVWIGLHDPKK.N RWHWSSGSLVSYK.S WHWSSGSLVSYK.S
16	29	5.52	10	128	34–44 45–54 90–106 90–107 110–122 111–122 123–148 149–157 151–163 158–163	ISCPEGTNAYR.S SYCYYFNEDR.E ESGTDDFNVWIGLHDPK.K ESGTDDFNVWIGLHDPKK.N RWHWSSGSLVSYK.S WHWSSGSLVSYK.S SWGIGAPSSVNPGYCVSLTSSTGFQK.W WKDVPCEDK.F DVPCEDKFSFVCK.F FSFVCK.F
17	29	5.73	9	144	34–44 45–54 90–106 90–107 110–122 111–122 123–148 151–163 158–163	ISCPEGTNAYR.S SYCYYFNEDR.E ESGTDDFNVWIGLHDPK.K ESGTDDFNVWIGLHDPKK.N RWHWSSGSLVSYK.S WHWSSGSLVSYK.S SWGIGAPSSVNPGYCVSLTSSTGFQK.W DVPCEDKFSFVCK.F FSFVCK.F
18	29	6.03	8	121	34–44 45–54 90–106 90–107 111–122 123–148 151–163 158–163	ISCPEGTNAYR.S SYCYYFNEDR.E ESGTDDFNVWIGLHDPK.K ESGTDDFNVWIGLHDPKK.N WHWSSGSLVSYK.S SWGIGAPSSVNPGYCVSLTSSTGFQK.W DVPCEDKFSFVCK.F FSFVCK.F
23	27	5.48	11	153	34–44 34–54 45–54 90–106 90–107 110–122 111–122 123–148 149–157 151–163 158–163	ISCPEGTNAYR.S ISCPEGTNAYRSYCYYFNEDR.E SYCYYFNEDR.E ESGTDDFNVWIGLHDPK.K ESGTDDFNVWIGLHDPKK.N RWHWSSGSLVSYK.S WHWSSGSLVSYK.S SWGIGAPSSVNPGYCVSLTSSTGFQK.W WKDVPCEDK.F DVPCEDKFSFVCK.F FSFVCK.F

### Gel electrophoresis analysis of pancreatic juice protein

To clarify the protein profile of pancreatic juice, the isolated proteins were assessed by SDS PAGE and 2D gel electrophoresis. Pancreatic juice from patients without stones showed 6 prominent bands at 71 kDa, 55 kDa, 30 kDa, 32 kDa, 23 kDa and 14 kDa (Fig. 2B). The proteins isolated from pancreatic juice were subjected to 2D gel electrophoresis with pH values ranging from 4 to 7 to assess the protein profile (Fig. 2D). Nine spots of proteins were seen at < 10.4 kDa with a PI range of 5–6.5. Fifteen spots of proteins were seen between 20 and 40 kDa with a PI range of 4–7. Five spots of proteins were at the 51 kDa position with a PI range of 4.5–6.5. A total of 29 spots with high intensities were excised, used for in-gel digestion and eventually identified by MALDI-TOF.

### MALDI-TOF of pancreatic juice

Twenty-nine in-Gel tryptic digested pancreatic juice spots were analysed using MALDI-MS. The resultant mass spectral data (PMF, Additional file 1: Fig. S6) were searched against the SWISS-PROT database using the MASCOT search engine. Of the twenty nine gel spots, eleven spots were identified as amylase, carboxypeptidase, trypsin, chymotrypsin and chymotrypsinogen. Out of the eleven identified spots, only one spot was identified as lithostathine (Reg1A).

### In vitro* precipitation of pancreatic juice*

**Fig. 3:**
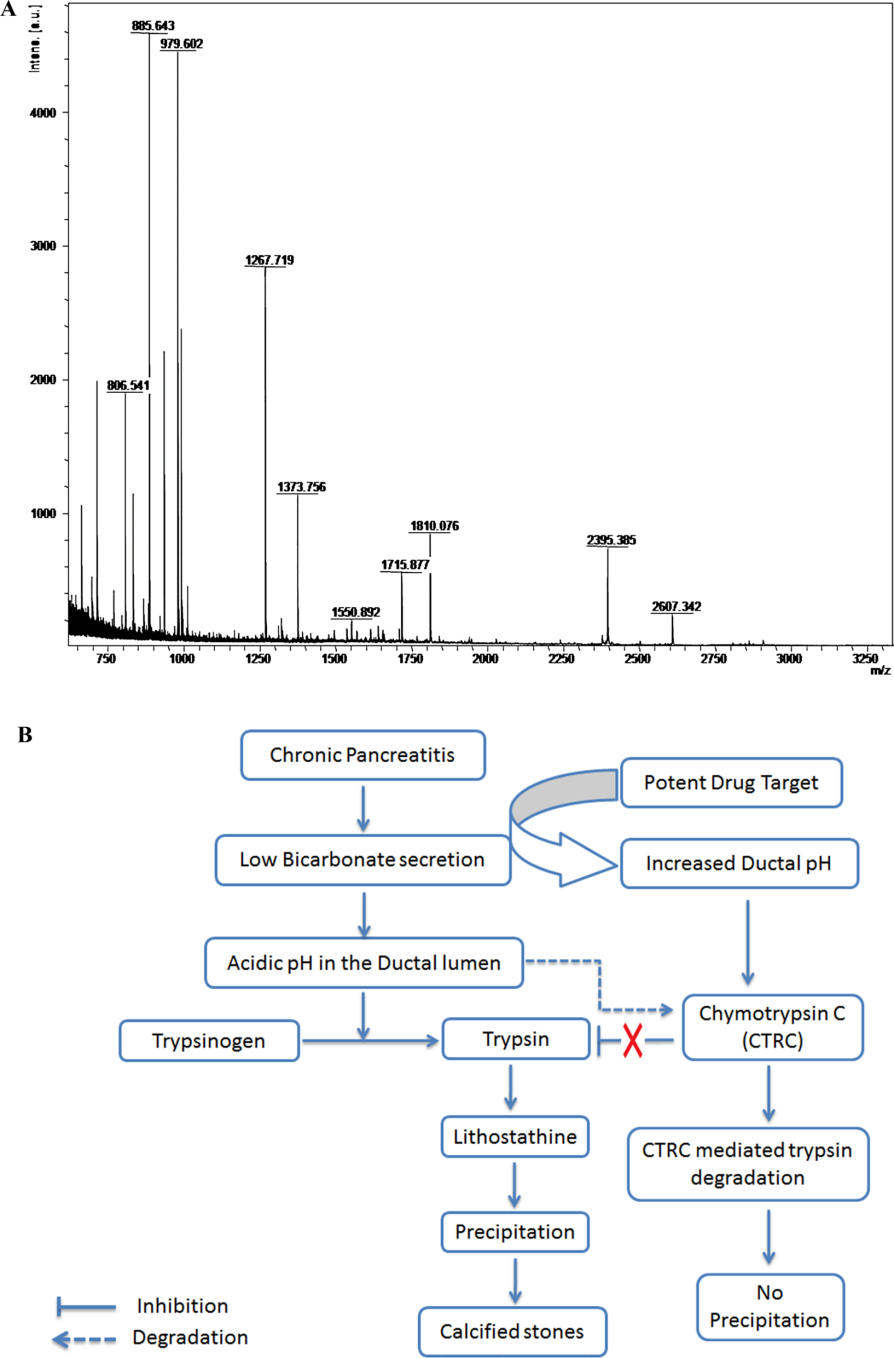
**A** Positive-mode MALDI-reflectron TOF mass spectra using HCCA as a matrix (1:1, v/v) of differentially expressed proteins observed at pH 6 and 7. **B** Hypothetical model of lithostathine precipitation and plug formation within the ductal lumen in CP patients

Protein precipitation was greater at trypsin concentrations between 8 and 12 µg/mL and incubation for 30 min. Therefore, the pancreatic juice was treated with 10 µg/mL trypsin, and its pH was varied from 4 to 8. After electrophoresis, protein degradation of protein was observed at pH 4, and few bands were visible at pH 5–8 (Additional file 1: Fig. S3). A single band was exclusively seen at pH 6 and 7 at the 18 kDa position. The protein was identified through excision of this band, gel digestion and MALDI-TOF of the extracted peptides (Fig. 3A). Four peptide fragments matched the sequence of chymotrypsin C (CTRC) with a molecular weight of 18 kDa and a score of 61. The sequences that matched CTRC were 113–119 (WNALLL. N), 152–162 (DYPCYVTGWGR. L) 173–188 (LQQGLQPVVDHATCSR. I), 189–195 (IDWWGFR.V).

### In silico analysis

**Fig. 4:**
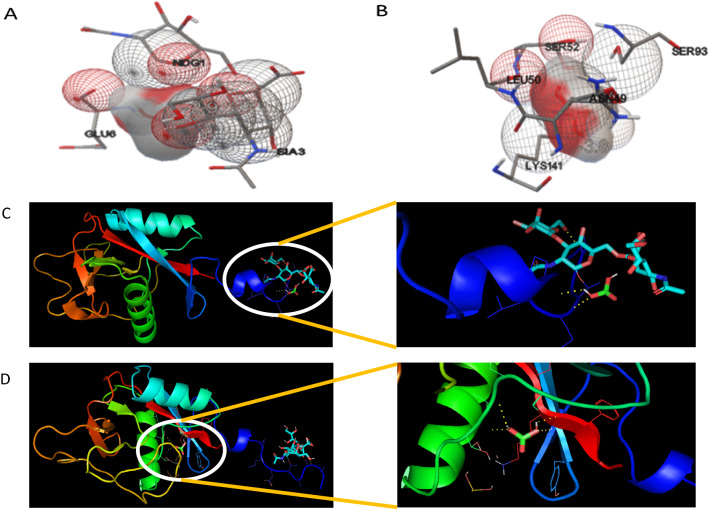
In silico analysis: Docking of native (**A** and **C**) and truncated (**B** and **D**) lithostathine with calcite molecules (green and red triangular molecules) present in the right corner of image C and in the centre of image D, showing the interaction of calcite molecules at different positions on lithostathine using AutoDock Vina and PyMOL software

Docking truncated lithostathine, which was identified in soft stones (protein plugs) and the native lithostathine (seen in pancreatic juice), with calcite molecule predicted a higher binding affinity for truncated lithostathine compared that of its native form. The binding energy of truncated lithostathine was − 3.8, whereas that of native lithostathine was − 2.9. β-D- Galactopyranose (NDG), sialic acid (SIA) and glutamic acid (Glu) at the 1, 3 and 6 positions were observed to interact with the calcite molecule of native lithostathine protein, whereas asparagine (Asn), leucine (Leu), 2 molecules of serine (Ser) and lysine (Lys) at the 49, 50, 52, 93, 141 positions, respectively, were predicted to interact with the truncated lithostathine protein. The interaction of both forms of lithostathine with calcite molecules is depicted in Fig. 4.

## Discussion

Although calcifications in the pancreatic duct and pancreatic parenchyma are a known phenomenon in CP 19–21, the mechanism of protein precipitation, calcifications and ductal obstruction are not completely understood. We conducted this study to unravel the events leading to lithostathine precipitation. Our study indicates that the process of protein plug formation begins with degradation of chymotrypsin C followed by precipitation of lithostathine and calcification resulting in the formation of stones.

In this study we found that the number of prediabetic and diabetic patients was greater among CP patients in whom all stones were calcified. This corroborates with earlier findings that calcifications increase susceptibility to diabetes [6]. Calcifications block the normal flow of pancreatic juice, and this blockage increases the intraductal pressure, which is known to cause greater inflammation [22]. It is known that inflammation enhances the risk that CP progresses to diabetes. Previous studies from our institute have also demonstrated that clearing calcified stones by ESWL decreased the incidence of diabetes in CP patients [5], suggesting that calcifications increase the risk of diabetes.

SDS PAGE was employed to evaluate the nature of proteins in protein plugs from CP patients, and we found two distinct bands. Hence, to achieve a better resolution and determine the complexity of the protein, 2D gel electrophoresis was carried out. Protein spots (23 spots) with different molecular weights ranging from < 10 to 29 kDa and protein spots of similar molecular weights at different PIs ranging from 4 to 7 on 2D gel electrophoresis were identified. This indicates the presence of isoforms of the same protein. Smaller and less intense spots could not be identified due to insufficient protein. MASCOT analysis of the chosen spots confirmed the protein to be lithostathine (14/23: 60.8%), suggesting that lithostathine is the dominant component of soft stones. It has been documented that 5 isoforms of lithostathine are present in pancreatic juice [9]. Our findings regarding the soft stones in CP patients are similar to the earlier results stating that the protein core of the stone is composed of lithostathine [23–25]. Besides identification, another cue observed after peptide mass fingerprinting of lithostathine protein was that all the protein spots lacked initial 11 amino acid sequence. This result provides the evidence of lithostathine being cleaved making it insoluble and precipitate within the pancreatic duct.

We then conducted 2D gel electrophoresis on pancreatic juice from CP patients without soft/calcified stones with an intension to profile the proteins of pancreatic juice. We identified only one spot as lithostathine and others as digestive enzymes, such as trypsin, chymotrypsin, chymotrypsinogen, amylase and carboxypeptidase. Whereas, all the 2D gel spots of protein plugs were identified solely as lithostathine. Further, we altered the pH of the pancreatic juice to produce an acidic environment at high concentrations of activated trypsin to mimic the CP environment. We observed protein precipitation at pH 6.0 and 7.0 with a trypsin concentration of 10 µg/mL. Surprisingly, the precipitated protein was identified to be chymotrypsin C by MALDI-TOF and not lithostathine. CTRC seems to be degraded as the molecular weight of precipitated protein was found to be 18 kDa but actual molecular weight of CTRC being 30 kDa. CTRC is known to play a crucial role in regulating the levels of trypsin via trypsinogen degradation [26–30]. Though the role of CTRC and lithostathine cleavage were reported earlier in independent studies, the link between them was not established with regard to protein plug formation and calcification.

In general, lithostathine is present in pancreatic juice of healthy individuals but its function still remains elusive. However, hypothetically, native form of lithostathine is known to prevent stone formation by inhibiting the growth of calcium carbonate crystals. However, the activity of cleaved lithostathine is not known. Hence, a simulation study was performed. Docking of cleaved (truncated) lithostathine and native lithostathine, was carried out with calcite molecule (supersaturated bicarbonate in pancreatic juice). Docking predictated that cleaved lithostathine exhibits a higher affinity to calcite (interpreted as per the binding energy generated by the software, which was high). The MALDI results in this study showed cleaved lithostathine, which lacked the initial 11 amino acid sequence. It can be suggested that due to the absence of the N-terminal 11 amino acid sequence, the calcium carbonate present in the supersaturated state in pancreatic juice tends to bind to the truncated lithostathine, leading to stone formation in CP patients.

A change in pancreatic ductal pH in CP, which occurs due to decreased ductal cell functions, might make chymotrypsin C (CTRC) susceptible to degradation by activated trypsin. Our data on genetic mutations in CTRC were also shown to be positively correlated with pancreatic ductal stone suggesting the role of CTRC in the protein precipitation and the consequences (data unpublished). Degraded CTRC cannot regulate the levels of activated trypsin within the pancreas [31, 32]. Hence, the activity of trypsin continues cleaving lithostathine, resulting in the precipitation and formation of insoluble aggregates. Cleaved lithostathine with higher binding affinity to calcium results in calcifications.

This study is a proof of concept study and has limitations. Further studies are needed to clarify the signalling mechanism that leads to CTRC inactivation in acidic environments at high concentrations of trypsin under in vivo conditions.

In conclusion, our results suggest that the acidic environment of the pancreatic ductal lumen resulting from decreased bicarbonate secretion leads to the degradation of chymotrypsin C. In this state, trypsin activity remains high, leading to the cleavage of proteins, including lithostathine, and protein aggregates form inside the ductal lumen as a result.

## Supplementary Information

**Additional file 1: Fig. S1.** Pictorial depiction of the study design. **Fig. S2.** Flowchart showing patients recruitment. **Fig. S3.** SDS-PAGE gel of Protein plugs extracted from different patients (1–6). M is the protein marker. **Fig. S4.** SDS-PAGE gel of Pancreatic juice treated with 10ug/ml trypsin at varying pH: lane1-pH 4, lane 2- pH 5, lane 3- pH 6, lane 4- pH 7, lane 5- pH 8, and lane 6- undigested Pancreatic Juice at pH 8. M is the protein marker. **Fig. S5.** Positive-mode MALDI-reflectron TOF mass spectra using CHCA as matrix (1:1, v/v) of lithostathine in soft stone/protein plugs. **Fig. S6.** Positive-mode MALDI-reflectron TOF mass spectra using CHCA as matrix (1:1, v/v) of lithostathine in Pancreatic Juice.

## Data Availability

The data analysed during the current study are available from the corresponding author on reasonable request.

## Abbreviations

CP: Chronic pancreatitis
PSP: Pancreatic stone protein
PD: Pancreatic duct
ERCP: Endoscopic retrograde cholangiopancreatography
EUS: Endoscopic ultrasound
MALDI-TOF: Matrix assisted laser desorption/ionization- time of flight
TCA: Trichloroacetic acid
PBS: Phosphate buffered saline
NaCl: Sodium chloride
SDS: Sodium dodecyl sulfate
PAGE: Poly acrylamide gel electrophoresis
2 D gel: Two-dimensional gel
DTT: Dithiothreitol
TFA: Trifluoroacetic acid
ACN: Acetonitrile
HCCA: α-Cyano-4-hydroxycinnamic acid
HbA1c: Hemoglobin A1C
PI: Isoelectric point
PMF: Peptide mass fingerprinting
Da: Dalton
KDa: Kilodalton
MS: Mass spectrometry
Reg1A: Regenerating islet-derived 1alpha
CTRC: Chymotrypsin C
