# Supplementary material for: Alterations in the pH of pancreatic juice are associated with chymotrypsin C inactivation and lithostathine precipitation in chronic pancreatitis patients: a proteomic approach
Source: Clin Proteomics. 2022 Dec 26;19:49. doi: 10.1186/s12014-022-09384-8 (PMC9791725; doi:10.1186/s12014-022-09384-8)
Supplement: Supplementary file 1 — Additional file 1: Fig. S1. Pictorial depiction of the study design. Fig. S2. Flowchart showing patients recruitment. Fig. S3. SDS-PAGE gel of Protein plugs extracted from different patients (1–6). M is the protein marker. Fig. S4. SDS-PAGE gel of Pancreatic juice treated with 10ug/ml trypsin at varying pH: lane1-pH 4, lane 2- pH 5, lane 3- pH 6, lane 4- pH 7, lane 5- pH 8, and lane 6- undigested Pancreatic Juice at pH 8. M is the protein marker. Fig. S5. Positive-mode MALDI-reflectron TOF mass spectra using CHCA as matrix (1:1, v/v) of lithostathine in soft stone/protein plugs. Fig. S6. Positive-mode MALDI-reflectron TOF mass spectra using CHCA as matrix (1:1, v/v) of lithostathine in Pancreatic Juice. [file 12014_2022_9384_MOESM1_ESM.docx]

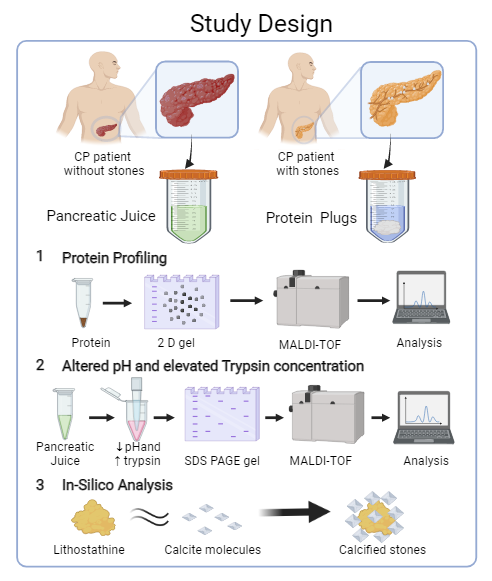
**Additional file Data**

Figure S1: Pictorial depiction of the study design.

**Patients:** Patients with documented CP were recruited from AIG Hospitals, Hyderabad during October 2018 to March 2021. All the eligible patients (n=2824) were screened for the presence of stones or protein plugs by either EUS or diagnostic ERCP. Symptomatic CP patients were advised to undergo ERCP for ductal decompression as a therapeutic protocol. Protein plugs were collected in PBS from patients (n=20 undergoing ERCP in the last 3 months of the study) who had soft/calcified stones. Pancreatic Juice was collected from CP patients (n=5) from the external drain, in a falcon tube containing a protease inhibitor and stored at -80^°^C (Figure 1). Patients with pancreatic neoplasms, Pregnancy, major medical comorbidities, confirmed malignancy, CP with suspected or confirmed malignancy and inability to give informed consent were excluded from the study. The study was approved by Institutional Ethics committee and all the participants had provided written informed consent.


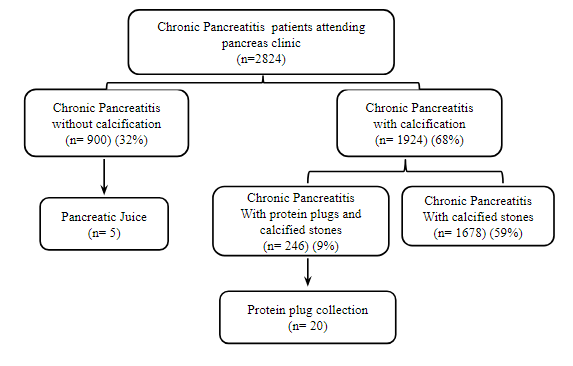
Figure S2: Flowchart showing patients recruitment.


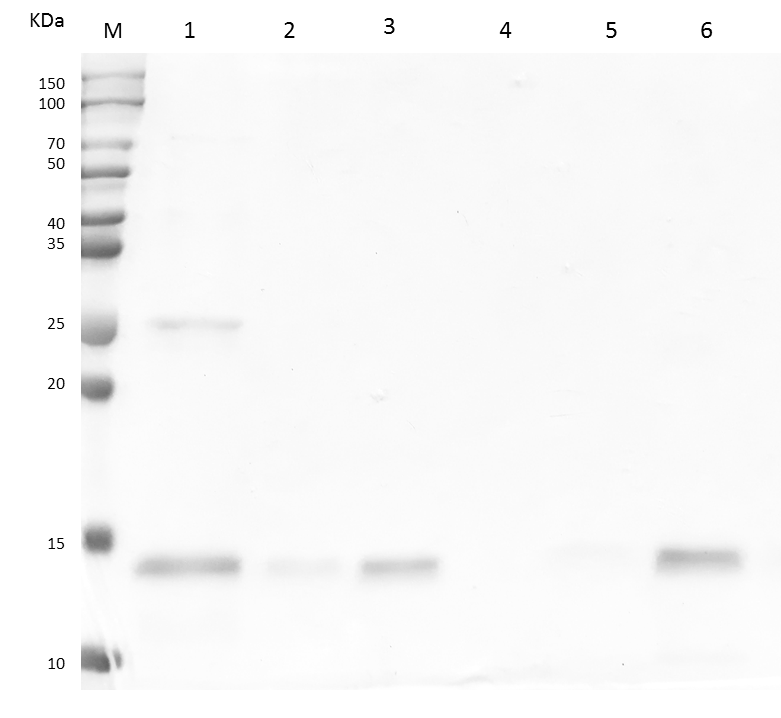
Figure S3: SDS-PAGE gel of Protein plugs extracted from different patients (1-6). M is the protein marker.


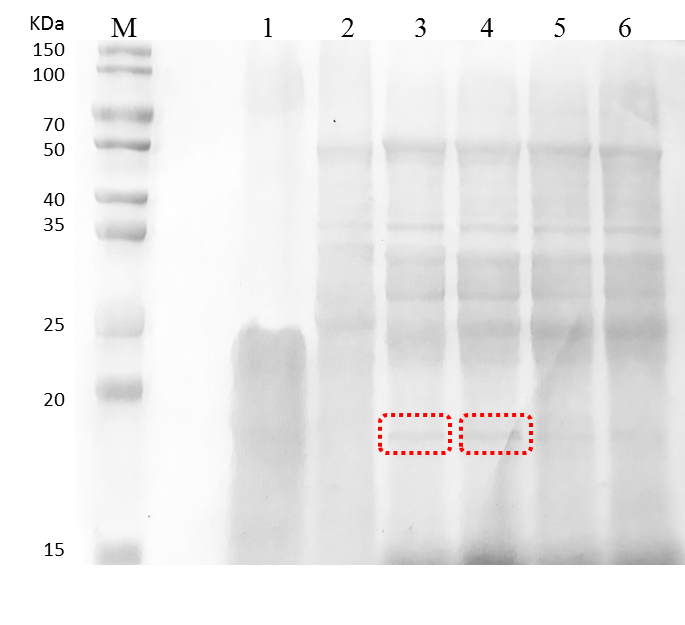


Figure S4: SDS-PAGE gel of Pancreatic juice treated with 10ug/ml trypsin at varying pH: lane1-pH 4, lane 2- pH 5, lane 3- pH 6, lane 4- pH 7, lane 5- pH 8, and lane 6- undigested Pancreatic Juice at pH 8. M is the protein marker.


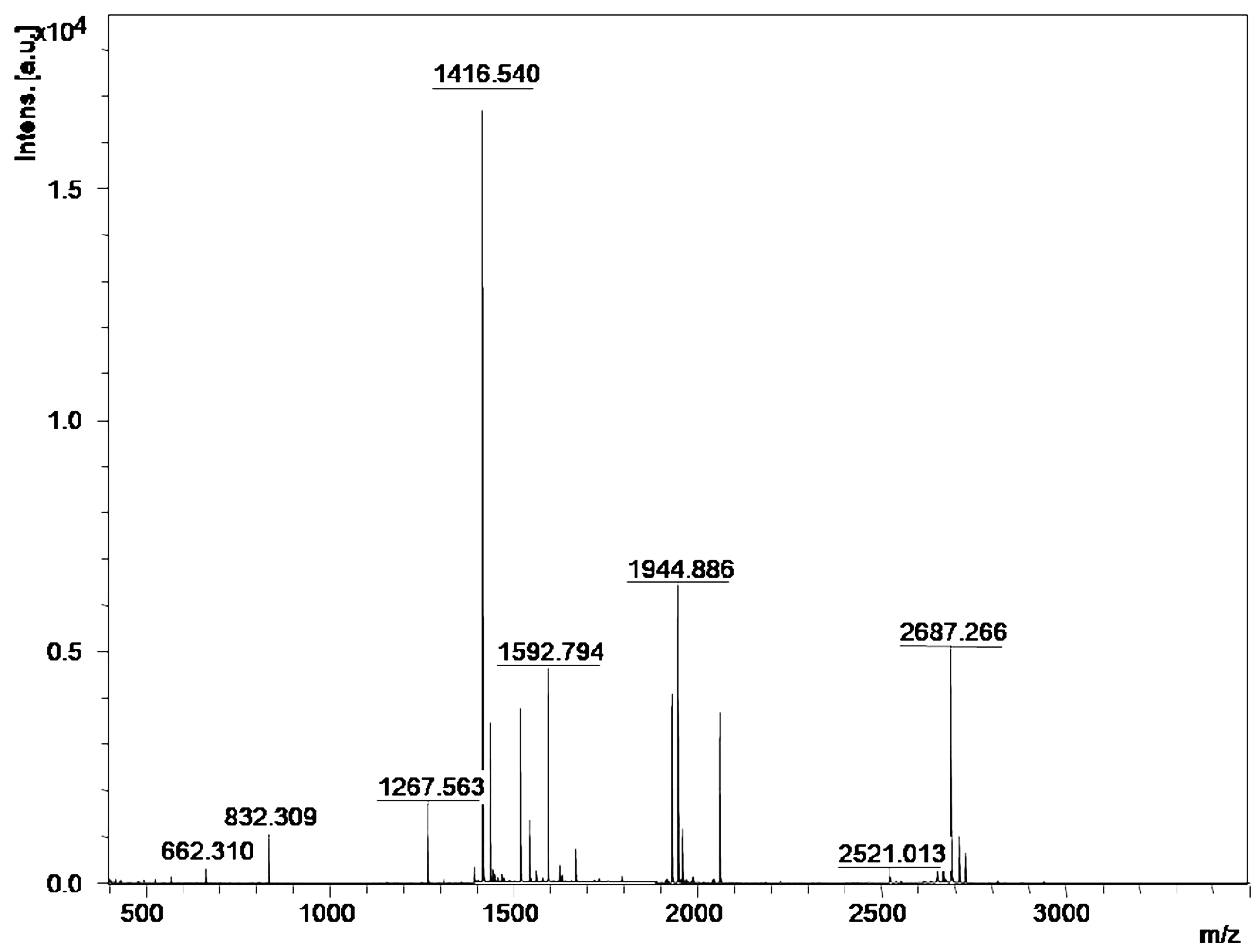


Figure S5: Positive-mode MALDI-reflectron TOF mass spectra using CHCA as matrix (1:1, v/v) of lithostathine in soft stone/protein plugs.


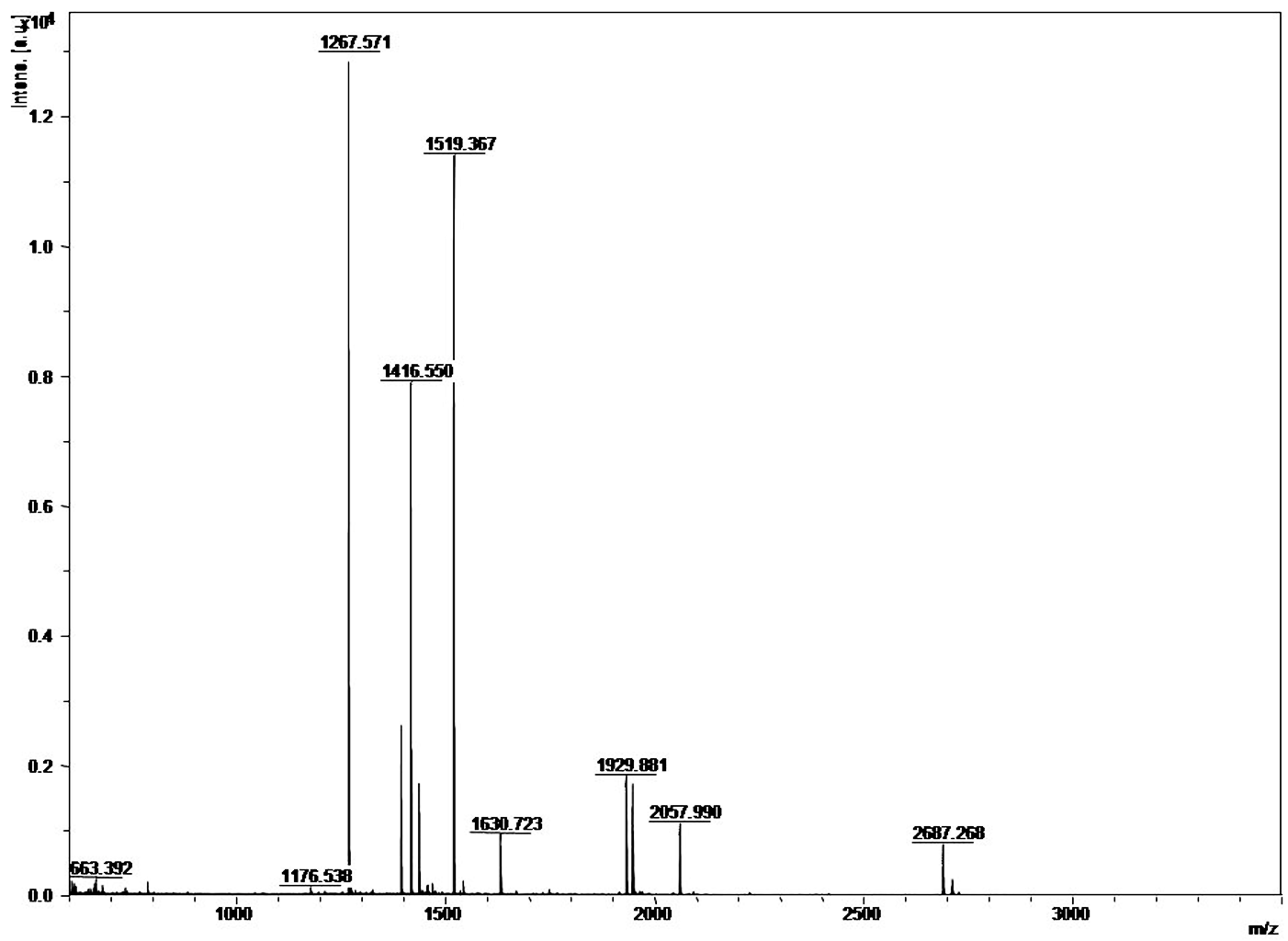


Figure S6: Positive-mode MALDI-reflectron TOF mass spectra using CHCA as matrix (1:1, v/v) of lithostathine in Pancreatic Juice.
